# A DBULSTM-Adaboost Model for Sea Surface Temperature Prediction

**DOI:** 10.7717/peerj-cs.1095

**Published:** 2022-09-30

**Authors:** Jiachen Yang, Jiaming Huo, Jingyi He, Taiqiu Xiao, Desheng Chen, Yang Li

**Affiliations:** Tianjin University, Tianjin, China

**Keywords:** DBULSTM, Adaboost, Deep learning, Sea surface temperature prediction, Marine environment

## Abstract

Sea surface temperature (SST) is an important parameter to measure the energy and heat balance of sea surface. The change of sea surface temperature has an important impact on the marine ecosystem, marine climate and marine environment. Therefore, sea surface temperature prediction has become an significant research direction in the field of ocean. This article proposes a DBULSTM-Adaboost model based on ensemble learning. The model is composed of Deep Bidirectional and Unidirectional Long Short Term Memory (DBULSTM) and Adaboost strong learner. DBULSTM can capture the forward and backward dependence of time series, and the DBULSTM model is integrated with Adaboost strong learner to reduce the variance and bias of prediction and realize the short and medium term prediction of SST at a single point scale. Experimental results show that the model can improve the accuracy and stability of SST prediction. Experiments on the East China Sea and South China Sea with different prediction lengths show that the model is almost superior to other classical models in different sea areas and at different prediction levels. Compared with full-connected LSTM (FC-LSTM) model, the root-mean-square error is reduced by about 0.1.

## Introduction

Sea surface temperature (SST) has played an indispensable role in the ocean atmosphere interaction, that is, the exchange of matter, energy, and momentum between the ocean and the atmosphere (Wentz et al., 2000; Sumner et al., 2003; Constable et al., 2014). As a result, changes in SST have incalculable impacts on global climate and marine ecosystem (Bouali, Sato & Polito, 2017; Cane et al., 1997; Meehl, 1990; Castro, Wick & Steele, 2016; Chaidez et al., 2017). SST predictions also have implications for applications related to the ocean, such as weather forecasting, fisheries and marine environmental protection. For example, SST changes affect precipitation distribution which further may lead to droughts and floods (Salles et al., 2016; Xiao et al., 2019). Therefore, it is critically necessary to predict dynamic changes of SST in the future to help people identify and prevent severe weather events and it is also of great significance for scientific research and applications (Chen et al., 2014).

The prediction method of sea surface temperature has been widely used and concerned, which has become a hot topic. These prediction methods are generally divided into three categories, including physics-based numerical methods, data-driven methods and methods combining the two Patil, Deo & Ravichandran (2016). Numerical methods in general is the use of the kinetics and thermodynamics equation to describe the SST changes, thus solving a series of differential equations, using physics and some important theory to establish the mathematical model of oceanography, described based on the physical conditions and processes of SST variation law. The methods shall be carried out in accordance with the change rule to SST forecast, and are usually used for areas with low resolution, rather than specific stations with high resolution (Stockdale, Balmaseda & Vidard, 2006). Krishnamurti et al. (2006) used integrated analysis and hyper-integration methods to predict SST anomalies, achieving SST prediction of the integrated model, and evaluating SST seasonal forecast and SST anomalies using deterministic and probabilistic methods. Noori et al. (2017) proposed a reduced order model based on feature vectors, which used 10 modes to accurately predict SST in the northwestern Indian Ocean, achieving simplicity and accuracy in predicting SST. However, numerical methods are usually complex and require a lot of computation and time, which makes it difficult to solve equations.

The data driven method is mainly used to predict the future ocean surface temperature from the perspective of data. The model is constructed by learning the relationships and patterns between historical SST data, and further using the learned relationship model to approximate the future SST data. The data-driven method is not as complex as the numerical method and is suitable for SST prediction of high resolution regions. Data-driven methods mainly involve traditional statistical data analysis methods, machine learning and artificial intelligence methods. Among them, traditional statistical techniques mainly include the Markov model (Xue, Leetmaa & Ji, 2000), empirical canonical correlation analysis and the regression model (Laepple & Jewson, 2007; Patil et al., 2013). Xue & Leetmaa (2000) used SST data and seasonal SST data to construct a Markov model in multivariate space, and realized the prediction of SST in the tropical Pacific. Collins, Reason & Tangang (2004) designed an empirical model using canonical correlation analysis containing the first 12 empirical orthogonal functions of Indian Ocean SST, and realized the prediction of sea level pressure and Indian Ocean SST in the next year under different historical time series lengths. Li-Wei, Fei & Jiang (2013) combined dynamic and statistical methods to construct a linear regression model based on the lag relationship between SST in tropical Indian Ocean and NINO 3.4 SST index, and realized the prediction of SST in equatorial eastern Pacific for 24 months.

The recurrent neural network (RNN) is an artificial neural network model used for time series processing, such as natural language processing, stock prediction and other time series problems, which can effectively extract time features from time series, so RNN can be used to predict SST (Graves, Mohamed & Hinton, 2013). However, RNN will have serious problems of gradient disappearance or gradient explosion after multiple gradient propagation in the training process, and it is difficult to train, so it cannot be applied in the field of long-term series prediction. SST prediction is highly dependent on marine data, so RNN will not perform well. In order to solve this long-term time-dependence problem, Hochreiter & Schmidhuber (1997) proposed LSTM network. LSTM is a special recursive neural network, which can remember longer time series information through its cyclic structure and gating mechanism, so as to obtain better prediction results. Zhang et al. (2017) used LSTM network and the network framework of full connection layer to realize short-term prediction of 1 and 3 days’ future SST as well as long-term prediction of weekly and monthly mean. As an improved variant of LSTM, the gated recurrent unit (GRU) has fewer gates and simpler structure while retaining the advantages of LSTM memory for long time series, which reduces training parameters. Therefore, GRU has higher computational efficiency and alleviates the phenomenon of over-fitting and under-fitting of network. A novel model is proposed by Yang et al. (2018), which essentially combines the temporal and spatial information to predict future SST values. This model solves the problem of ignoring spatial factors in temperature prediction. Dey & Salem (2017) used GRU and full connection layer to capture the time characteristic rule of SST series, and realized the accuracy and stability prediction of monthly average and seasonal SST in the Bohai Sea region. Xie et al. (2019) designed a mechanism of the adaptive mechanism based on GRU helped and attention to predict sea surface temperature, the mechanism of using GRU helped to capture the changes of sea surface temperature in the static and dynamic effect chain is used to capture the dynamic effect, the sea surface temperature coding and early prediction of the future sea surface temperatures, and therefore solves the long scale dependency problem. Xiao et al. (2019) proposed a machine learning method that combined the long and short-term memory deep recursive neural network model with Adaboost algorithm to predict short-term and medium-term daily SST. Feng, Sun & Li (2021) used time-domain convolutional network to achieve short-term small-scale SST prediction of the Indian Ocean. Han et al. (2019) used convolutional neural network method to achieve regional prediction of SST, sea surface height and ocean salinity in the Pacific. Park et al. (2019) used the proposed temperature prediction model to fill in the missing data to recover the missing SST data. After filling in all the missing data, the model based on LSTM was retrained to achieve the prediction of short-term SST in the future 6, 12 and 24 h. Zhang, Geng & Yan (2020) proposed a multi-layer convolutional long and short-term memory model to predict 3d ocean temperature. The model is composed of convolutional neural network and long and short-term memory, which can predict horizontal and vertical changes of ocean temperature below about 2,000 m. Yang et al. (2018) added attention mechanism to weight the output of each step of LSTM model to adjust the prediction results, which improves the prediction accuracy of the approach. Li et al. (2022) utilized the probability entropy and distance entropy to evaluate the data, which has reference significance for data preprocessing.

The existing studies on SST prediction only consider the one-way time series dependence and ignore the reverse time series information when capturing the time features. In order to make full use of all the information contained in the SST time series as well as the periodicity and regularity of the series, this article not only considers the forward and reverse dependence of the time series, but also obtains a prediction model with lower bias and variance. A deep bidirectional and unidirectional long short term memory (DBULSTM-Adaboost) model based on ensemble learning is proposed. The model realizes the prediction of short and medium SST at single point scale and improves the accuracy and stability of prediction.

The contribution of this article can be summarized in the following two aspects: (1) In this article, we apply bidirectional long short term memory (BLSTM) to SST prediction for the first time. An deep bidirectional and unidirectional long short term memory (DBULSTM) is proposed. Stacking BLSTM and LSTM constructs DBULSTM deep architecture, further integrates forward and backward dependence, and captures all feature information in time series. (2) The DBULSTM model and Adaboost strong ensemble learning model are integrated with an average strategy to obtain a prediction model with low bias and low variance. The SST model has been used to predict SST at different scales on two real data sets in the East China Sea and the South China Sea. Experiments verify the effectiveness of the DBULSTM-Adaboost model in obtaining the forward and backward dependence relationship, and it is almost superior to other baseline models in different sea areas and different prediction lengths, showing the stability of prediction. The rest of the article is organized as follows. “Research Area and Data” introduces the marine area selected in this study and the SST dataset used in the experiment. The “Methods” proposes the novel STAGCN model for SST prediction in detail. “Experiments and Discussion” visualize the experimental results and make correlative discussions. Finally, “Conclusion” gives the conclusion of the article.

## Research area and data

**Figure 1:**
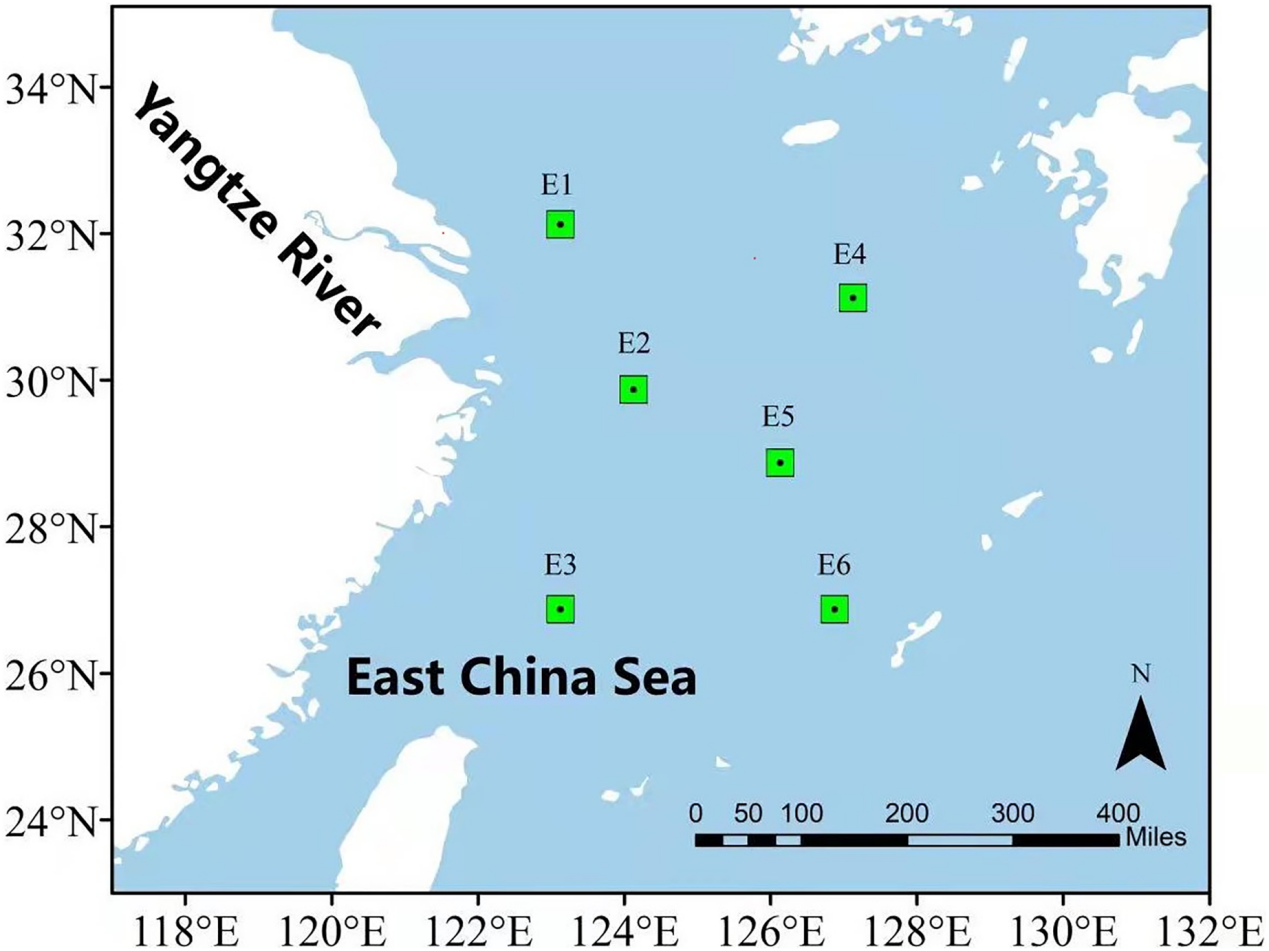
Location of the East China Sea research area.

**Figure 2:**
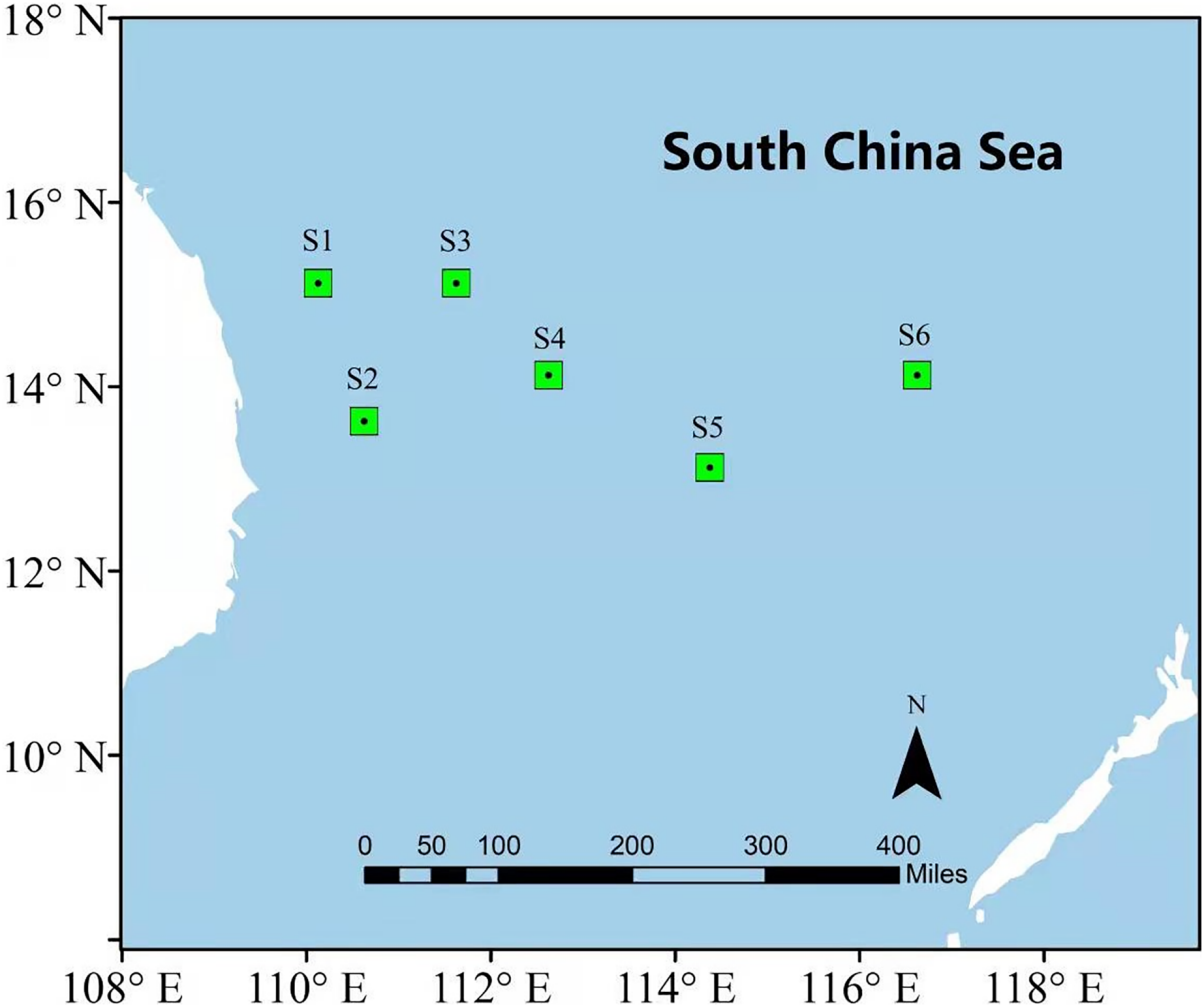
Location of the South China Sea research area.

In order to illustrate the prediction performance of the proposed DBULSTM-Adaboost model, two research regions are selected in this section, namely, the East China Sea and the South China Sea. The East China Sea is rich in natural resources and is the confluence of several rivers. With the Bohai Sea in the north and Taiwan Strait in the south, it is a strategic maritime area for China, Japan, South Korea and other countries. The South China Sea is one of China’s three marginal seas, rich in mineral resources and an important checkpoint for maritime resource transportation. Therefore, it is very important to study the dynamic changes of SST data in the East China Sea and the South China Sea for China’s maritime transportation and the production and life of people in neighboring countries. In this section, six representative points in the East China Sea and South China Sea are selected as research points, and each location point is marked by a green dot box, as shown in Figs. 1 and 2. The six points in the East China Sea are denoted as E1 to E6, wherein E1 to E3 are close to the coast, E4 to E6 are selected as locations far from the coast, and the coordinate positions of the six points are E1 (32.125°N,123.125°E), E2 (29.875°N,124.125°E), E3 (26.875°N,123.125°E), E4 (31.125°N,127.125°E), E5 (28.875°N,126.125°E), E6 (26.875°N,126.875°E) respectively. The geographic coordinates of the six points in the South China Sea are denoted as S1 (15.125°N,110.125°E), S2 (13.625°N,110.625°E), S3 (15.125°N,111.625°E), S4 (14.125°N,112.625°E), S5 (13.125°N,114.375°E), S6 (14.125°N,116.625°E).

The data used in this study are noaa’s 1/4° daily best interpolated sea surface temperature. In order to reduce the error of measurement data, the national Oceanic and Atmospheric Administration platform adopts interpolation method to complete the missing values, which makes the SST spatial data more complete. In this section, the SST data sets of six locations in the East China Sea from 1982/1/1 to 2019/12/31 and the SST data sets of six locations in the South China Sea from 2000/1/1 to 2019/12/31 were selected to verify the prediction performance of DBULSTM-Adaboost model and verify the superiority of DBULSTM-Adaboost model over other baseline models in predicting performance on two datasets. Before using data for prediction, we eliminated abnormal values and use interpolation method to deal with the missing values. Tables 1 and 2 show the statistical characteristics of SST at the selected six coordinate points in the East China Sea and the South China Sea. It can be seen from the table that the average sea surface temperature at the east China Sea position point E1 has similar average values. In the South China Sea data set, S1, S2, S3 and S4 have similar mean values, while S5 and S6 have similar mean values and variances. E1 has the smallest temperature value on 1/4, 1/2, 3/4 data set, which may be because E1 is closest to the mainland and the ocean depth is shallower than other points in the ocean. The data set has rich features suitable for verifying the validity of the proposed prediction model.

**Table 1:** Statistical characteristics of SST at six coordinate points in the East China Sea.

Location	Mean	Stv.	Min	25%	50%	75%	Max
E1	17.813	6.258	6.030	11.700	18.400	23.460	30.650
E2	20.689	5.282	10.500	15.589	20.880	25.640	30.470
E3	24.022	3.179	16.800	21.189	23.859	26.920	30.840
E4	21.629	4.363	14.070	17.570	21.010	25.539	31.449
E5	23.015	3.960	15.360	19.300	22.869	26.619	31.080
E6	25.448	2.662	19.000	23.060	25.269	27.910	31.289

**Table 2:** Statistical characteristics of SST at six coordinate points in the South China Sea.

Location	Mean	Stv.	Min	25%	50%	75%	Max
S1	27.515	1.919	22.369	25.930	27.820	29.179	31.260
S2	27.683	1.544	23.619	26.439	27.900	28.910	31.109
S3	27.986	1.299	24.150	27.029	28.090	28.949	31.429
S4	27.841	1.460	23.490	26.699	28.039	28.949	31.590
S5	28.188	1.212	24.320	27.310	28.220	29.039	31.939
S6	28.385	1.204	24.500	27.510	28.449	29.199	32.070

## Methods

Stacking LSTM networks with multiple hidden layers to build a deep architecture can gradually build a higher level of sequence data representation and improve the performance of the model (Zeyer et al., 2017; LeCun, Bengio & Hinton, 2015). The deep LSTM architecture is a network of stacked LSTM hidden layers, where the output of one LSTM hidden layer serves as the input for subsequent LSTM hidden layers. This section adopts a DBULSTM model, which is a deep architecture of stacked multi-layer BLSTM and LSTM network, to achieve long-term and short-term prediction of SST. In the DBULSTM model, BLSTM is taken as the first layer of the network, and SST information is input into BLSTM to obtain time forward and backward correlation from time series and learn more characteristic information. At the same time, to further obtain forward and backward dependencies, multi-layer BLSTM networks can be stacked to improve the predictive performance of the model. When predicting future SST values, DBULSTM architecture adopts LSTM layer capturing forward dependence as the last layer of the model. LSTM layer uses the time series characteristic information output by the upper BLSTM network to perform forward iteration calculation in time order, and finally generates the predicted SST values in the future time period.

**Figure 3:**
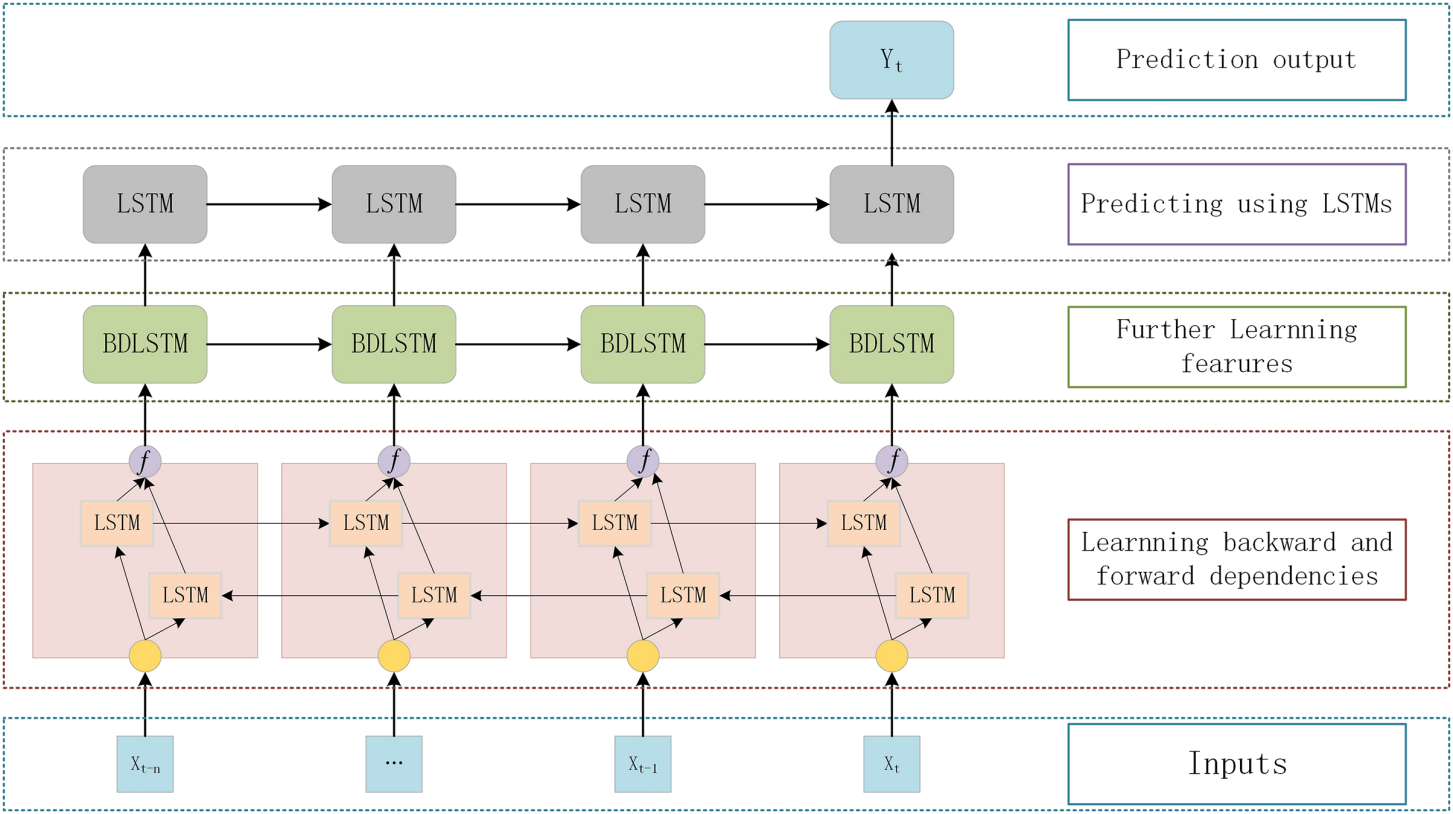
The DBULSTM model.

The specific structure of the deep architecture of stacked multi-layer BLSTM and one-way LSTM networks proposed in this section is shown in Fig. 3. Each DBULSTM model contains the BLSTM network as the first layer of the model and the LSTM network as the last layer of the model. In order to make full use of the input SST time series data and learn more comprehensive characteristic information, one or multiple BLSTM layers can be stacked in the middle of the DBULSTM model. DBULSTM model takes time series data as input and captures the forward and backward correlation dependencies in the series, which can predict SST values of one time step in the future and multiple future time steps based on historical data. Therefore, DBULSTM retains information from the future and uses a combination of two hidden states to save past and future information at any point in time. In the structure of DBULSTM, the LSTM layer is before the output layer, which is a kind of gated recurrent neural networks. It has a good performance on sequence-based tasks with long-term dependencies, which can handle the gradient exploding in traditional RNNs. The LSTM cell has three gates namely input gate, forget gate, and output gate, that control the information flow through the neural network (Ge et al., 2019). The formulas of the three gates and cell input state are as follows:

(1) $${i_t} = \delta \left( {{W_i}{x_t} + {U_i}{h_{t - 1}} + {b_i}} \right),$$

(2) $${f_t} = \delta \left( {{W_f}{x_t} + {U_f}{h_{t - 1}} + {b_f}} \right),$$

(3) $${o_t} = \delta \left( {{W_o}{x_t} + {U_o}{h_{t - 1}} + {b_o}} \right),$$

(4) $${\rm }{\tilde C_t} = \tanh \, \left( {{W_C}{x_t} + {U_C}{h_{t - 1}} + {b_C}} \right)$$

where ${i_t},{f_t},{o_t},{\rm }{\tilde C_t}$ represent the input gate, the forget gate, the output gate and the cell input state respectively, ${W_i},{W_f},{W_o}$ and ${W_C}$ are the weight matrices transforming input ${x_t}$ to the three gates and cell input state, ${U_i},{U_f},{U_o}$ and ${U_C}$ are the weight matrices transforming previous cell output state ${h_{t - 1}}$ to the three gates and cell input state, ${b_i},{b_f},{b_o}$ and ${b_C}$ are the bais vectors of the three gates and cell input state. $\delta$ denotes the gate activation function and $\tanh$ denotes the hyperbolic tangent function. The cell output state and the hidden layer are calculated as Eqs.(5) and (6):

(5) $${C_t} = {i_t}*{\rm }{\tilde C_t} + {f_t}*{C_{t - 1}}$$

(6) $${h_t} = {o_t}*\tanh \, \left( {{C_t}} \right)$$

In time series prediction, the prediction performance of the model can be improved by integrating different models, proving the advantages of the hybrid model prediction mechanism (Messias et al., 2016). Adaboost is a powerful integrated learning method, which is used for low-bias prediction tasks and is not easy to be over-fitted in the training process, and has good generalization performance (Bartlett et al., 1998). Moreover, both DBULSTM model and Adaboost model have low bias errors. Therefore, In order to obtain a prediction model with lower deviation and variance, DBULSTM model and Adaboost average strategy are integrated in this article to predict SST in the short and medium term to improve the accuracy and stability of prediction (Perrone, 1993).

**Figure 4:**
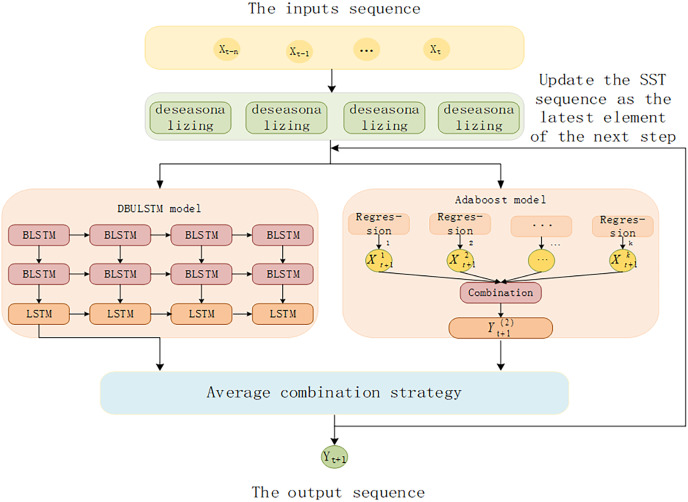
The architecture of DBULSTM and Adaboost integration model.

The architecture of DBULSTM-Adaboost integration model proposed in this article is shown in Fig. 4. Firstly, the SST time series data are divided into data sample sets through the continuous movement of time window, and the data set is used as the input of DBULSTM model and Adaboost model to train the two models respectively. Then, the predicted results generated by DBULSTM model and Adaboost model are averaged and then used as the latest element of the next long SST time series to update the series, which is used to predict the next SST value. The error between the predicted value and the real value is used to update the weight of multiple weak learning devices. Multiple step size prediction tasks can be realized by repeating the above steps.

## Experiments and discussion

### Experiment setup

In this article, we will propose the DBULSTM-Adaboost integration model and compare it with four different models, including linear support vector regression model (SVR), LSTM model, GRU model and BLSTM model. All experiments in this section are performed in the Ubuntu20.04 server environment. The proposed DBULSTM-Adaboost integration model is implemented using Tensorflow1.14 (GPU version). Other baseline models such as the LSTM model, BLSTM model, DBULSTM model, Adaboost model and SVR model are implemented by Statsmodels 0.12.2 library and SciKit-learn 0.24.1 library respectively. We took 80% of the data as training samples, and the remaining 20% as test samples. The number of hidden units in LSTM is 20, and the initial learning rate is 0.001. Adam was used as the optimizer of the training model. In order to prove the prediction performance of the DBULSTM-Adaboost integrated model proposed in this study, root mean square error (RMSE), mean absolute error (MAE) and mean absolute percentage error (MAPE) are adopted in this section to measure the prediction performance of the STAGCN model and other baseline models for SEA surface temperature, which are defined as follows:

(7) $$RMSE = \sqrt {\displaystyle{1 \over T}\sum\limits_{t = 1}^T {{{({y_t} - {{\hat y}_t})}^2}} }$$

(8) $$MSE = \displaystyle{1 \over T}\sum\limits_{t = 1}^T | {y_t} - {\hat y_t}|$$

(9) $$MAPE = \displaystyle{{100\% } \over T}\sum\limits_{i = 1}^T {\left| {\displaystyle{{{{\hat y}_i} - {y_i}} \over {{y_i}}}} \right|}$$

where, ${y_t}$ and ${\hat y_t}$ respectively represent the real sea surface temperature value and the predicted sea surface temperature value at the first moment, and *T* is the number of time samples. In the prediction results of SST by the model, the lower the RMSE and MAE values are, the higher the fitting degree between the predicted value and the real value is, the lower the prediction error of the model is, and the better the prediction performance is.

Before inputting the SST time series into the DBULSTM model and Adaboost model for training, we removed the seasonal features and normalized the input series. The seasonal component of time series data sets is a recurring cycle, which may obscure the prediction results and affect the prediction model. From sea surface temperature time series in the process of removing the seasonal component called seasonal adjustment, the process can make the relationship between the input and output sequence more clearly, to explore the trend of time series and periodic, and compared with the predict directly using the primary data has a higher accuracy, and thus improve the performance of the model prediction. First, SST data with seasonal components removed were used as inputs to DBULSTM and Adaboost models to train models for prediction. Second, seasonal characteristics are added to the forecast results to obtain the final forecast. At the same time, in order to accelerate the convergence of the model, the deseasonal time series at each position are normalized to make the data more centralized and improve the prediction accuracy of the model.

At present, the methods to remove seasonal components from time series have been very mature. There are two main methods: one is to use the difference characteristics, the other is to model the seasonal characteristics. In this section, the latter is adopted to directly model the seasonal components in the SST time series, and then remove the seasonal components from the original time series. In the time series of SEA surface temperature, the seasonal component is a polynomial curve with fixed period and amplitude, so the curve fitting can be used to approximate the seasonal component. First, a new time series is constructed by taking the original time series as independent variable (day as unit). The fitting is then performed by calling the Polyfit () function in the NUMPY package, which uses X-axis values (time index), Y-axis values (sea surface temperature observations) and the order of the polynomial to fit the new time series, where the order of the polynomial controls the complexity of the fitting curve by the number of control items, The model form obtained is shown in Eq. (10):

(10) $$y = \sum\limits_{i = 0}^n {{a_i}} {x^i}$$

where, *n* is the polynomial order of the fitting curve, ${a_i}$ represents the fitting coefficient of each time variable in the polynomial, and *y* is the polynomial curve of the fitting seasonal component finally obtained. In this section, the order value of the polynomial is set to 6 to model the seasonal components in the historical and future time series, obtain the seasonal factor change curve in the time series, remove the seasonal components from the series data, make the relationship between the input SST time series and the predicted results more clear, and improve the accuracy of prediction.

**Figure 5:**
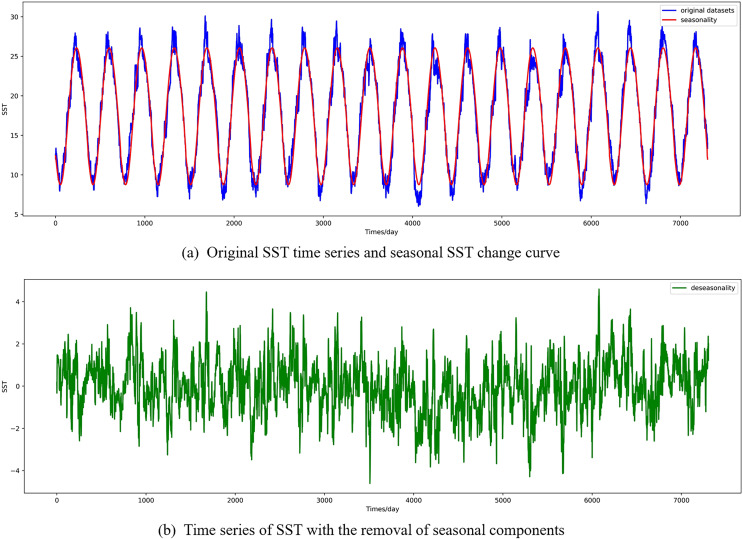
Visualized results of SST de-seasonalization at E1 in the East China Sea region. (A) Original SST time series and seasonal SST change curve, (B) time series of SST with the removal of seasonal components.

**Figure 6:**
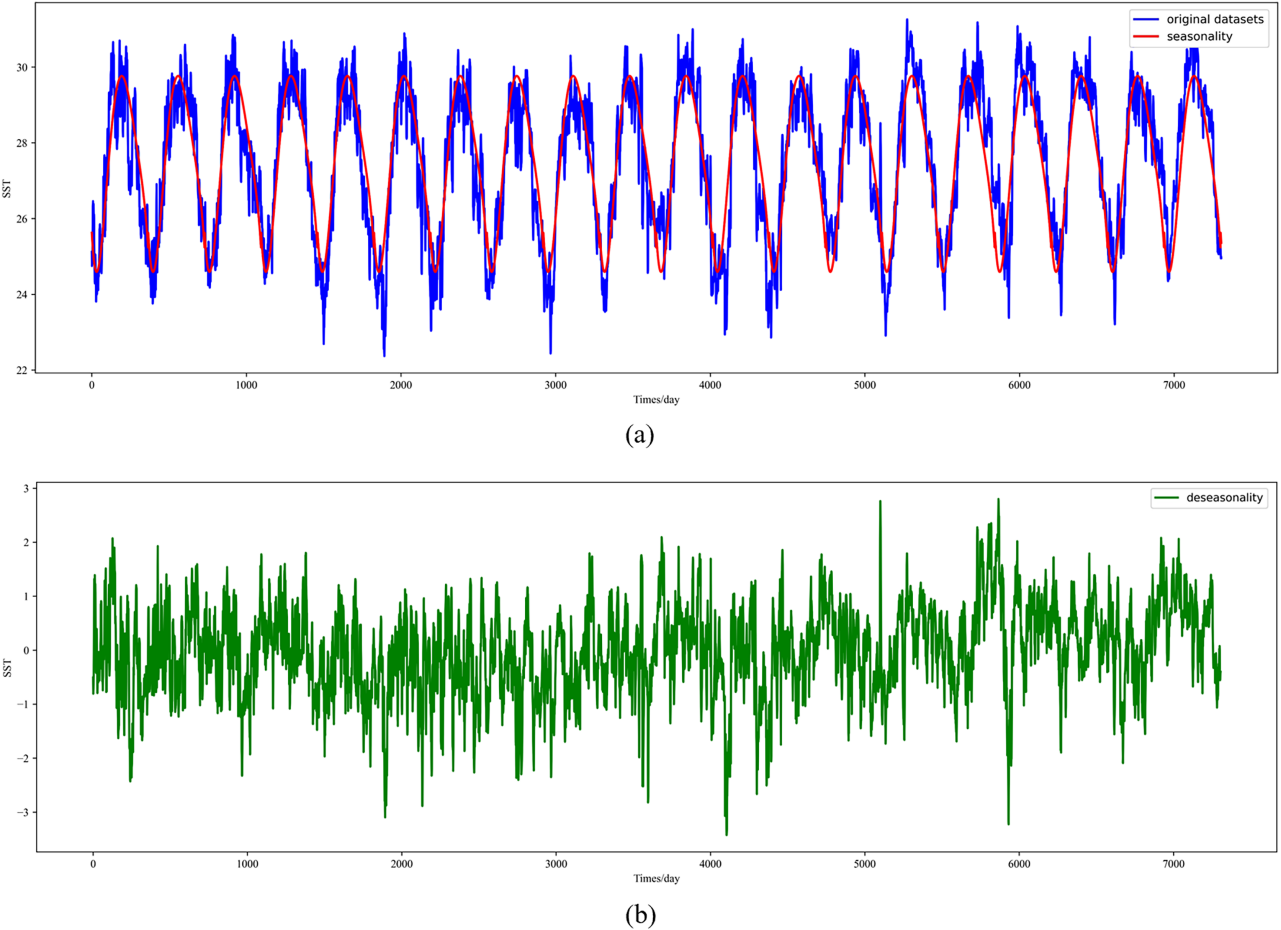
Visualized results of SST de-seasonalization at S1 in the South China Sea region. (A) Original SST time series and seasonal SST change curve, (B) time series of SST with the removal of seasonal components.

In this section, the diseasonal results of the ocean surface temperature time series of E1 in the East China Sea study location site and S1 in the South China Sea study location site are shown in Figs. 5 and 6. As can be seen from the Figs. 5 and 6, the SST time series has obvious seasonality, and the change range of SST value after removing seasonality is smaller, which is conducive to capture the change relationship between sequence value and predicted value and accelerate the model convergence.

Before the time series of sea surface temperature with no seasonal components are input into model training, they are normalized to the range [0–1]. Specifically, each time series is divided by the maximum value in the series for normalization processing, as shown in Eq. (11):

(11) $${x_{norm}} = \displaystyle{{{x_{dess}}} \over {{x_{max}}}}$$

where ${x_{norm}}$ represents the result of normalization, ${x_{dess}}$ is the time series of removing seasonal components, and ${x_{max}}$ represents the maximum value in the time series. The normalized preprocessing of the input data can accelerate the convergence of the model and improve the efficiency of the training model.

### Experiment results and discussion

**Figure 7:**
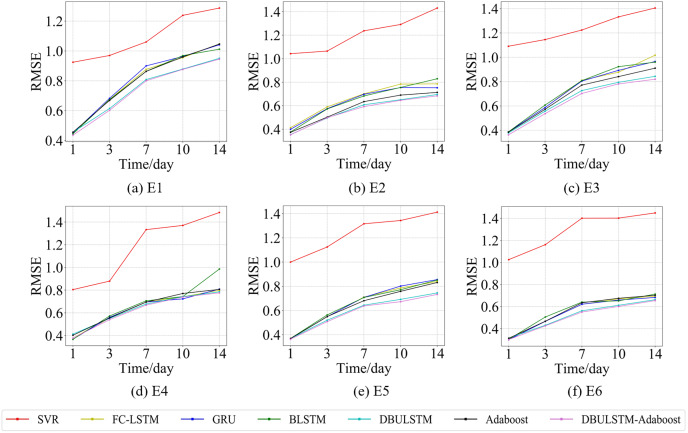
The RMSE changes for each model on the dataset of the East China Sea. (A) The RMSE changes of point E1 in the East China Sea. (B) The RMSE changes of point E2 in the East China Sea. (C) The RMSE changes of point E3 in the East China Sea. (D) The RMSE changes of point E4 in the East China Sea. (E) The RMSE changes of point E5 in the East China Sea. (F) The RMSE changes of point E6 in the East China Sea.

**Figure 8:**
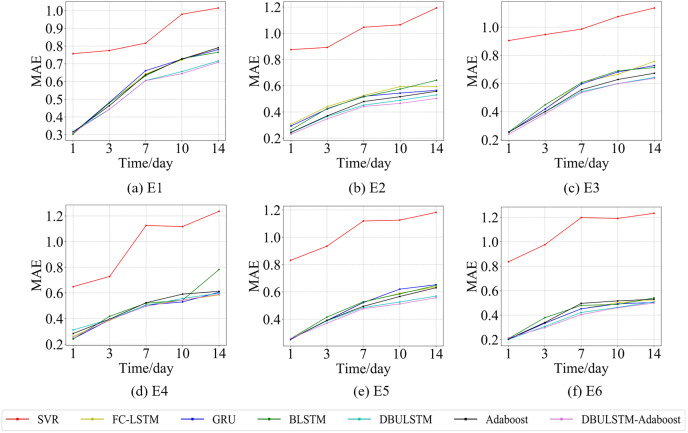
The MAE changes for each model on the dataset of the East China Sea. (A) The MAE changes of point E1 in the East China Sea. (B) The MAE changes of point E2 in the East China Sea. (C) The MAE changes of point E3 in the East China Sea. (D) The MAE changes of point E4 in the East China Sea. (E) The MAE changes of point E5 in the East China Sea. (F) The MAE of point E6 in the East China Sea.

**Figure 9:**
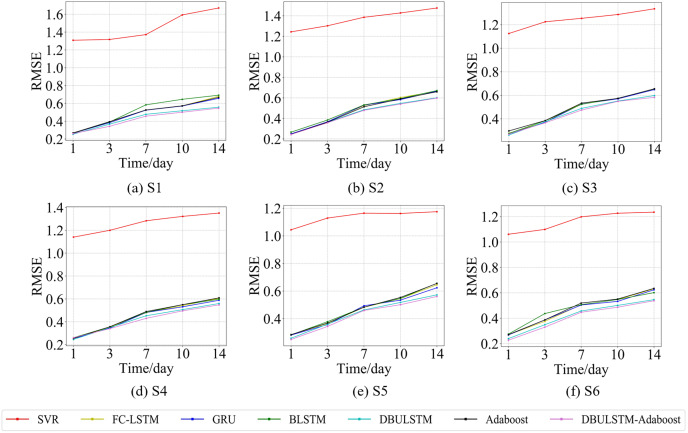
The RMSE changes for each model on the dataset of the South China Sea. (A) The RMSE changes of point S1 in the South China Sea. (B) The RMSE changes of point S2 in the South China Sea. (C) The RMSE changes of point S3 in the South China Sea. (D) The RMSE changes of point S4 in the South China Sea. (E) The RMSE changes of point S5 in the South China Sea. (F) The RMSE changes of point S6 in the South China Sea.

**Figure 10:**
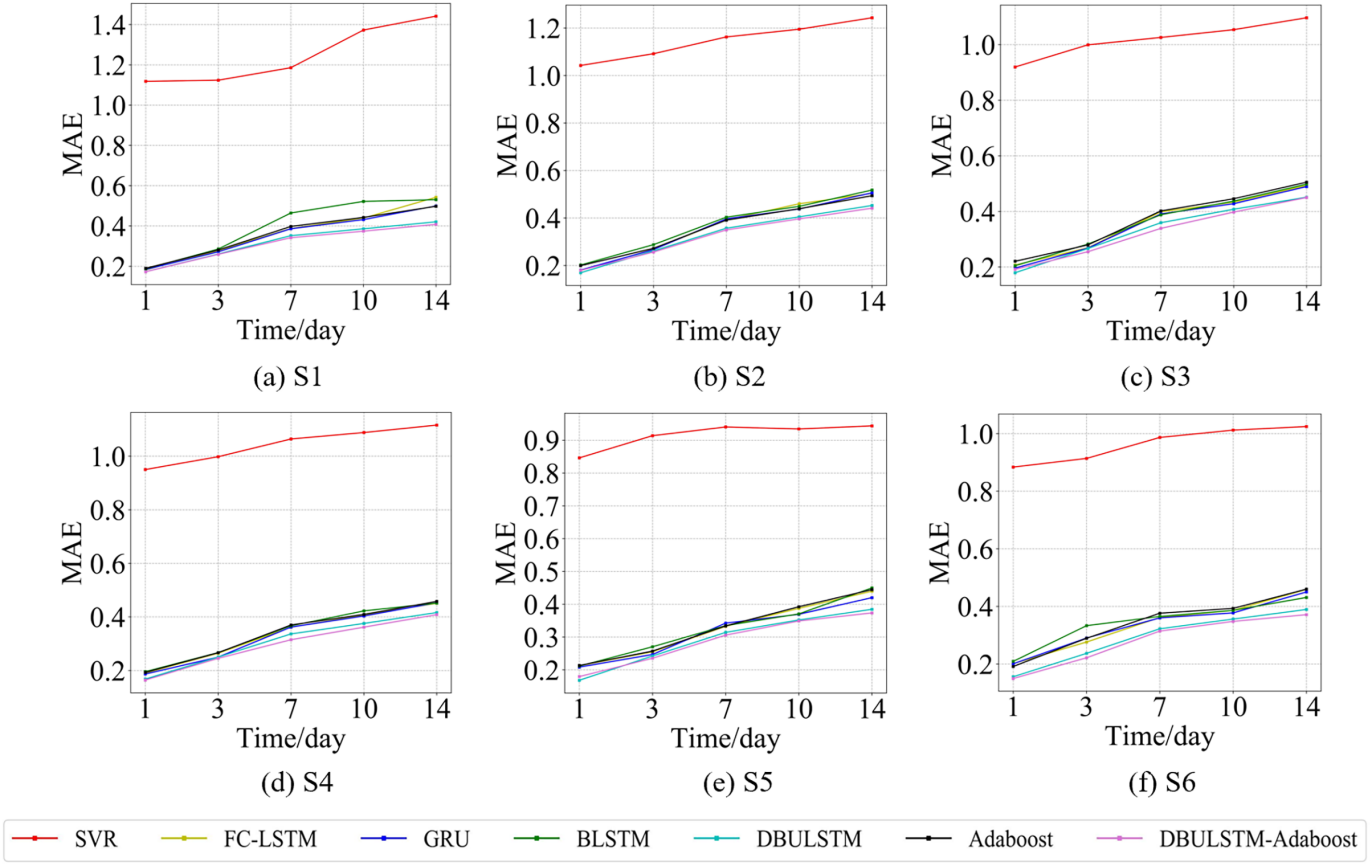
The MAE changes for each model on the dataset of the South China Sea. (A) The MAE changes of point S1 in the South China Sea. (B) The MAE changes of point S2 in the South China Sea. (C) The MAE changes of point S3 in the South China Sea. (D) The MAE changes of point S4 in the South China Sea. (E) The MAE changes of point S5 in the South China Sea. (F) The MAE changes of point S6 in the South China Sea.

In order to prove the prediction ability of the DBULSTM-Adaboost integration model proposed in this article, first of all, this section visualized the prediction performance of the DBULSTM-Adaboost integration model, four baseline models, DBULSTM model and Adaboost model under different prediction lengths. The predicted root mean square error (RMSE) and mean absolute error (MAE) were analyzed. Secondly, the improvement degree of prediction performance of DBULSTM-Adaboost integrated model, DBULSTM model and Adaboost model under different prediction lengths is analyzed. Thirdly, the prediction results of DBULSTM model, multi-layer BLSTM model and multi-layer LSTM model are analyzed. Finally, in order to better illustrate the prediction performance of DBULSTM-Adaboost integrated model, the prediction results of position point E1 on the East China Sea dataset and position point S1 on the South China Sea dataset are visualized in Figs. 7–10.

Figures 7 and 9 show the variation curves of RMSE of prediction error of DBULSTM-Adaboost integration model and other baseline models at different prediction lengths at each location of the East China Sea and South China Sea datasets, respectively. The variation curves of MAE of corresponding datasets are shown in Figs. 8 and 10. As shown in the figures, the prediction errors of all models gradually increase with the increase of the prediction length. For different prediction levels, the proposed DBULSTM-Adaboost model, LSTM model, BLSTM model, Adaboost integrated model and DBULSTM model all have better prediction performance than SVR model, which may be because SVR model is difficult to capture the time correlation in time series. As can be seen from the figure, DBULSTM-Adaboost model’s prediction error changes are almost at the lowest level compared with other baseline models on the two data sets, indicating that for different location points and at different prediction levels, The prediction performance of DBULSTM-Adaboost integration model is better than SVR model, LSTM model, BLSTM model, Adaboost integration model and DBULSTM model. The prediction performance of DBULSTM model at different prediction lengths is not always better than that of other baseline models, and the prediction error of Adaboost integrated model is not lower than that of other baseline models at all position points. At all position points, at different prediction levels, The prediction effect of DBULSTM model and Adaboost model is not fixed all the time, so the integration of DBULSTM model and Adaboost model can improve the accuracy of SST prediction. Although the prediction effect of DBULSTM-Adaboost model is not much improved compared with DBULSTM, it is better than other baseline models, and has certain advantages in the accuracy and stability of prediction at different prediction levels. Figures 8 and 10 show MAE variation curves of the predicted results under different predicted lengths at the locations of the east China Sea and South China Sea datasets respectively, and their variation trends are similar to RMSE variation on the corresponding datasets.

In this article, error RMSE of DBULSTM-Adaboost model, Adaboost model and DBULSTM model are compared at six locations in the East China Sea. The results are shown in Table 3, in which D-Adaboost represents DBULSTM-Adaboost model. The lowest RMSE of the prediction error at each location point at different prediction levels is shown in bold. As can be seen from the table, DBULSTM-Adaboost model achieves the lowest prediction error and the best prediction effect at all position points except for the prediction results of E2 and E3 in the future 1 day. For example, at position point E1, the RMSE value of DBULSTM-Adaboost model decreases by about 0.04 and 0.05 respectively relative to the Adaboost model for the prediction of the next 1 and 7 days. The RMSE value of DBULSTM-Adaboost model decreased by about 0.02 and 0.01, respectively.

**Table 3:** The RMSE of DBULSTM-Adaboost model compare with DBULSTM and Adaboost.

Time	Model	E1	E2	E3	E4	E5	E6
1 day	Adaboost	0.4553	0.3733	0.3845	0.4021	0.3681	0.3124
	DBULSTM	0.4551	0.3533	0.3782	0.4126	0.3677	0.3141
	D-Adaboost	0.4353	0.3518	0.3625	0.3773	0.3623	0.3003
3 days	Adaboost	0.6684	0.5027	0.5727	0.5548	0.5495	0.4673
	DBULSTM	0.6144	0.4956	0.5567	0.5501	0.5198	0.4305
	D-Adaboost	0.6020	0.4978	0.5364	0.5398	0.5079	0.4251
7 days	Adaboost	0.8629	0.6346	0.7713	0.6947	0.6808	0.6344
	DBULSTM	0.8081	0.6078	0.7264	0.6772	0.6437	0.5619
	D-Adaboost	0.7998	0.5917	0.7027	0.6676	0.6371	0.5502
10 days	Adaboost	0.9596	0.6900	0.8413	0.7704	0.7579	0.6751
	DBULSTM	0.8784	0.6508	0.7945	0.7419	0.6919	0.6116
	D-Adaboost	0.8662	0.6456	0.7810	0.7388	0.6724	0.6011
14 days	Adaboost	1.0460	0.7129	0.9106	0.8063	0.8313	0.7023
	DBULSTM	0.9595	0.6952	0.8434	0.7847	0.7451	0.6622
	D-Adaboost	0.9448	0.6835	0.8197	0.7757	0.7324	0.6539

In order to explore the prediction performance of Multiply LSTM (M_LSTM) and stacked BLSTM (Multiply BLSTM, M_BLSTM), DBULSTM model, M_LSTM model and M_BLSTM model are used to predict the SST of the next day at E1 in the East China Sea. The results of RMSE, MAE and MAPE of the prediction errors of the three models under different network layers are shown in Table 4. M = 0 indicates that the DBULSTM model contains one layer of BLSTM and one layer of LSTM. As can be seen from the table, the prediction errors of almost all models gradually increase with the increase of network depth, except for the LSTM model, when M = 4, the error tends to decline. With the increase of network layers, DBULSTM model has better prediction results than M_LSTM model and M_BLSTM model, although the prediction errors RMSE and MAE values gradually increase. For example, compared with M_LSTM model, when M = 1, 2, 3, 4, the prediction error RMSE of DBULSTM model decreases by about 0.02, 0.04, 0.064, 0.026 respectively. Compared with M_BLSTM model, when M = 1, 2, 3, 4, the prediction error RMSE of DBULSTM model decreases by about 0.018, 0.032, 0.0357, 0.026, respectively. Compared with other baseline models, DBULSTM model reduces the prediction error and improves the accuracy of SST prediction, and has certain advantages of prediction performance. At the same time, when DBULSTM model has no intermediate layer, the lowest prediction error is achieved, and RMSE, MAE and MAPE reach the minimum value of 0.4241, 0.2899 and 1.6274% respectively. It can also be seen from Table 4 that when the number of layers increases from 1 to 3, the prediction error of BLSTM model is lower than that of LSTM model, indicating that the USE of BLSTM model can capture the dependency before and after and improve the accuracy of prediction. For BLSTM model and LSTM model, when the number of intermediate layers is 1, RMSE achieves the lowest value of 0.4447 and 0.4439 respectively, indicating that single-layer BLSTM and LSTM model can capture features well.

**Table 4:** SST prediction errors of DBULSTM model and LSTM-based model at different network depths. Here DBULSTM refers to 1 layer BLSTM + M layer BLSTM + 1 layer LSTM.

Model	Metrics	The layer of model				
		M = 0	M = 1	M = 2	M = 3	M = 4
M_LSTM	RMSE	N/A	0.4447	0.4665	0.4902	0.4593
	MAE	N/A	0.3025	0.3288	0.3635	0.3211
	MAPE	N/A	1.6982%	1.8458%	2.2406%	1.8026%
M_BLSTM	RMSE	N/A	0.4439	0.4598	0.4615	0.4840
	MAE	N/A	0.3034	0.3249	0.3267	0.3474
	MAPE	N/A	1.7033%	1.8240%	1.8341%	1.9503%
DBULSTM	RMSE	0.4241	0.4255	0.4271	0.4258	0.4330
	MAE	0.2899	0.2952	0.2982	0.2932	0.3067
	MAPE	1.6274%	1.6572%	1.6741%	1.6460%	1.7218%

**Figure 11:**
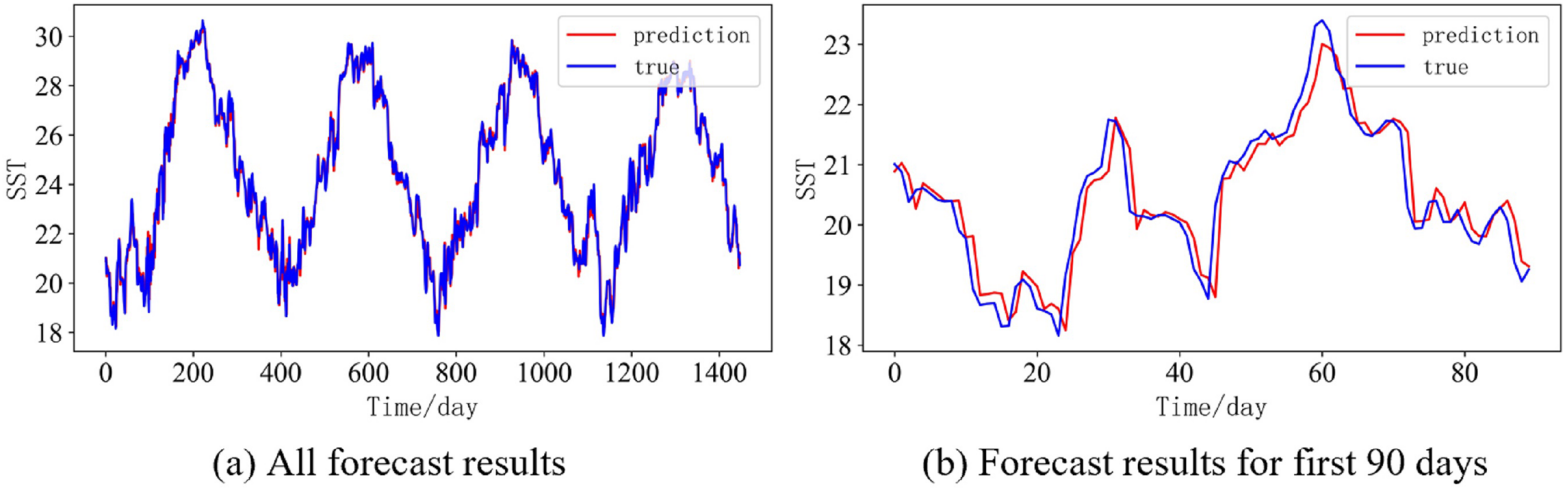
Prediction results of DBULSTM-Adaboost model when the prediction length is 1 day. (A) All forecast results, (B) Forecast results for first 90 days.

**Figure 12:**
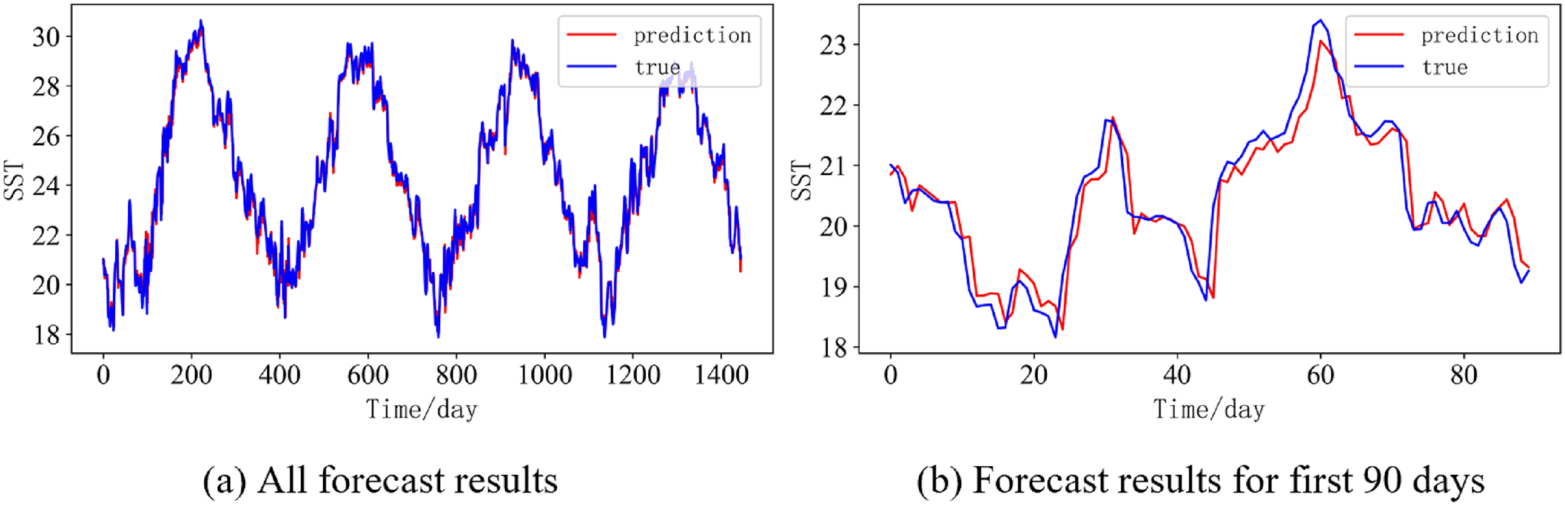
Prediction results of DBULSTM-Adaboost model when the prediction length is 3 day. (A) All forecast results, (B) Forecast results for first 90 days.

**Figure 13:**
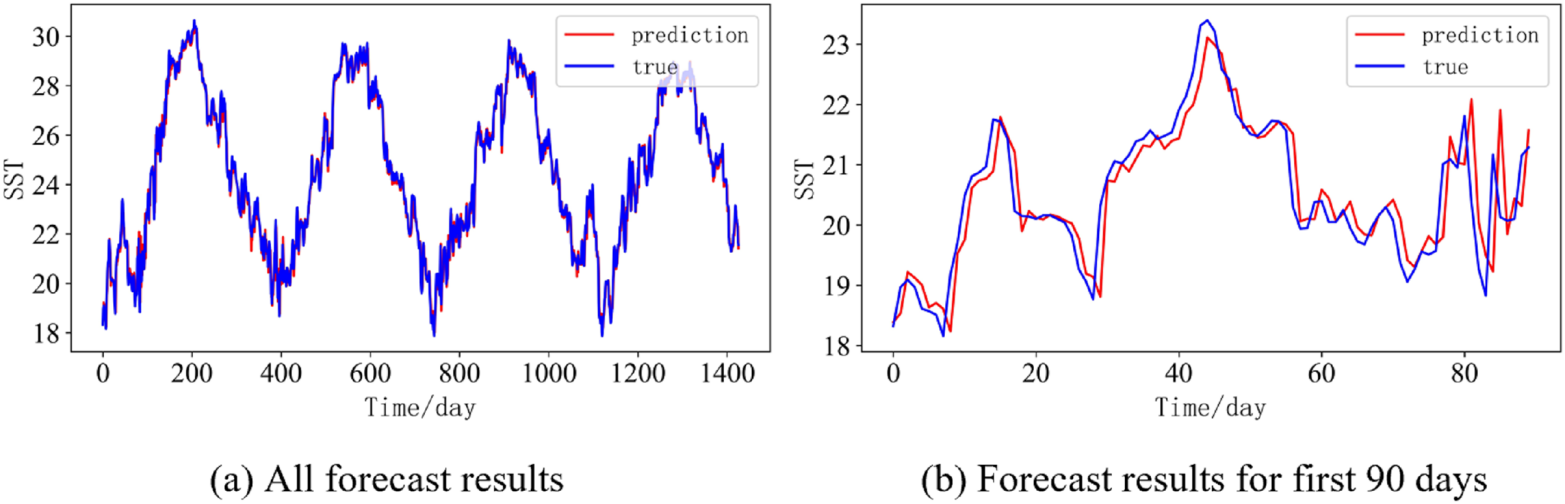
Prediction results of DBULSTM-Adaboost model when the prediction length is 7 day. (A) All forecast results, (B) Forecast results for first 90 days.

**Figure 14:**
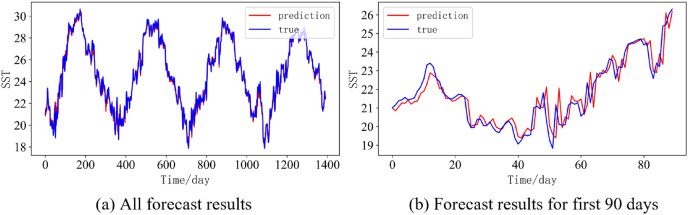
Prediction results of DBULSTM-Adaboost model when the prediction length is 10 day. (A) All forecast results, (B) Forecast results for first 90 days.

**Figure 15:**
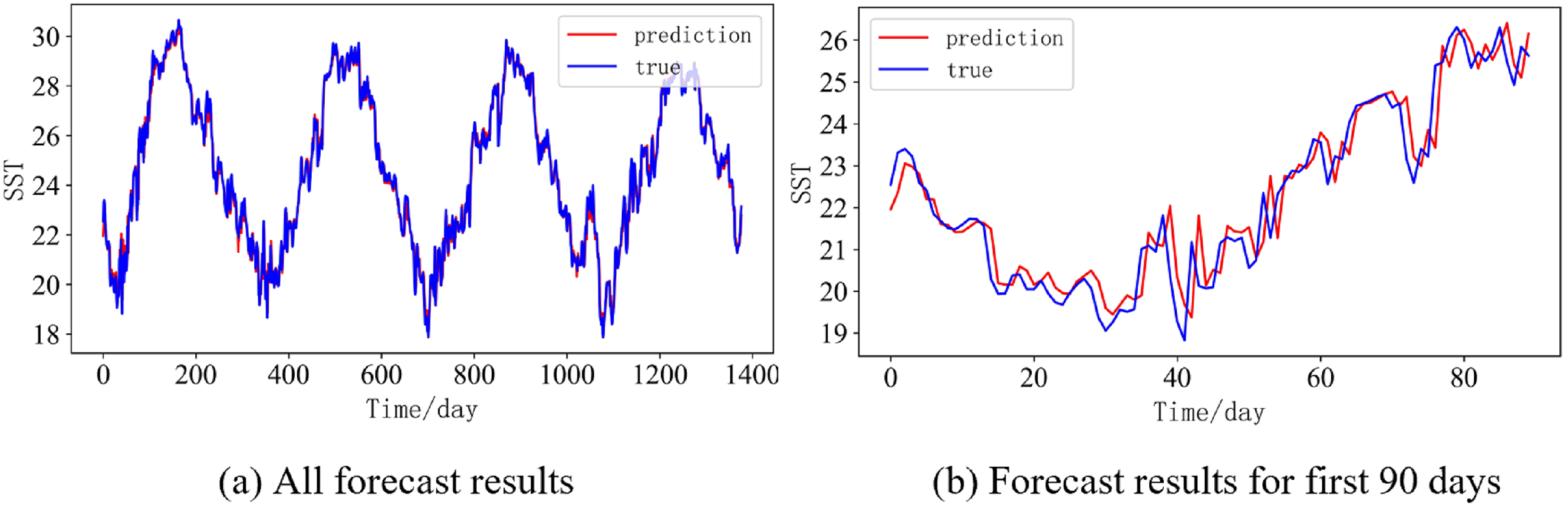
Prediction results of DBULSTM-Adaboost model when the prediction length is 14 day. (A) All forecast results, (B) Forecast results for first 90 days.

In order to better illustrate the prediction performance of DBULSTM-Adaboost model, we select location point E3 in the East China Sea data set to visually display the prediction results of the model on the test set. Figures 11–15 respectively show all the predicted results of The DBULSTM-Adaboost model for the next 1, 3, 7, 10 and 14 days and the predicted change curves for consecutive 90 days at this location. The prediction results of DBULSTM-Adaboost model show that the model can obtain good prediction results under different prediction lengths, and the predicted value fits the real value well. And the deviation of prediction is low. Although the prediction of the model at the maximum value is biased and the prediction performance decreases with the increase of the prediction length, this does not affect the accuracy and stability of the model prediction. DBULSTM-Adaboost model integrates DBULSTM and Adaboost, and constructs DBULSTM model by stacking deep BLSTM and LSTM. BLSTM obtains forward and backward dependence in time series, and LSTM as the last layer of model captures more characteristic information from SST series. Meanwhile, DBULSTM model is integrated with Adaboost to reduce the prediction variance and deviation value, improve the prediction performance, and realize the short and medium term prediction of single point SST.

## Conclusion

In this article, a DBULSTM-Adaboost model based on ensemble learning is proposed to realize the short and medium term prediction of ocean surface temperature at a single point scale. In the DBULSTM-Adaboost model, stacked BLSTM and unidirectional LSTM are used to build the DBULSTM model to capture the forward and backward dependence of the SST time series, and make full use of the features in the time series to improve the performance of prediction. The proposed model performs better than the commonly used prediction model at present. Meanwhile, DBULSTM model and Adaboost strong ensemble learning model are integrated with an average strategy to obtain a prediction model with low deviation and low variance, which can achieve accurate prediction of SST at single point scale. In the experiments, DBULSTM-Adaboost model is used to predict the short and medium term SST based on two real data sets of the East China Sea and South China Sea. The results show that DBULSTM-Adaboost model is stable and accurate.

## Supplemental Information

10.7717/peerj-cs.1095/supp-1 Supplemental Information 1 Part 1 of the raw data and code.

10.7717/peerj-cs.1095/supp-2 Supplemental Information 2 Part 2 of the raw data and code.

10.7717/peerj-cs.1095/supp-3 Supplemental Information 3 Part 3 of the raw data and code.

10.7717/peerj-cs.1095/supp-4 Supplemental Information 4 Part 4 of the raw data and code.

10.7717/peerj-cs.1095/supp-5 Supplemental Information 5 Part 5 of the raw data and code.

10.7717/peerj-cs.1095/supp-6 Supplemental Information 6 Part 6 of the raw data and code.

## Additional Information and Declarations

### Data Availability

The following information was supplied regarding data availability: The raw data and the code are available in the Supplemental Files.
